# Association Between Mental Health Outcomes and Changes in Lifestyle Behavior Index Among Saudi Adults 16 Weeks After COVID-19 Pandemic Lockdown Release

**DOI:** 10.3389/fpubh.2021.728117

**Published:** 2022-02-04

**Authors:** Nesrin Kamal Abd El-Fatah, Abdalsalam Abdullah Alshehri, Fatma H. Alsulami, Norah Alasmari, Nermin A. Osman

**Affiliations:** ^1^Department of Nutrition, High Institute of Public Health, Alexandria University, Alexandria, Egypt; ^2^Joint Postgraduate Training Program for Preventive Medicine, Ministry of Health, Taif, Saudi Arabia; ^3^Preventive Medicine and Public Health, Ministry of Health, Taif, Saudi Arabia; ^4^Department of Biomedical Informatics and Medical Statistics, Medical Research Institute, Alexandria University, Alexandria, Egypt

**Keywords:** depression, post-lockdown, COVID, composite lifestyle score, Saudi Arabia, social determinants, anxiety, stress

## Abstract

**Background:**

The current (coronavirus disease 2019 [COVID-19]) pandemic is still uncontrolled with associated dramatic changes in daily lifestyle activities. Evidence for studying the impact of these health behavior changes on our mental health is limited. Therefore, this study aimed to estimate the prevalence of psychological distresses and assess their influence by the change in the composite lifestyle behaviors before the COVID-19 pandemic till 16 weeks after the lockdown release in Saudi Arabia.

**Methods:**

This cross-sectional study was conducted between October 10 and 31, 2020 by posting an online survey on social media platforms (WhatsApp and Twitter) to collect data on participants' sociodemographic, lifestyle behaviors, and mental health aspects using a validated Arabic version of the short-form version of the Depression Anxiety Stress Scales-21 (DASS-21).

**Results:**

A total of 363 responded to the questionnaire. The mean age was 36.26 ± 8.54 years, and 238 (65.6%) were men. Depression, stress, and anxiety were reported in 37.5, 26.7, and 16.5% of the participants, respectively. Negative lifestyle behavioral changes were significantly associated with stress and anxiety (*p* < 0.05). Logistic regression revealed that financial distress and history of psychiatric illnesses were common significant factors for developing the psychological distresses.

**Conclusion:**

Throughout the post-lockdown stage of the COVID-19 outbreak in Saudi Arabia, there was an evidence of psychological distresses among the adults. Negative health-related changes are directly linked with increased psychological distress. Effective health promotion strategies directed toward adopting and maintaining positive change in the composite health behaviors are crucial.

## Introduction

The new coronavirus (coronavirus disease 2019 [COVID-19]) was first spotted in November 2019 in Wuhan. The World Health Organization's International Health Regulations 2005 declared this outbreak as a Public Health Emergency of International Concern and in March 2020, the WHO reported COVID-19 as a pandemic. According to the WHO weekly report from October 11 to 18, 2020, the incidence of globally reported new COVID-19 cases has continued to accelerate (over 2.4 million new cases), while the incidence of new deaths has remained relatively stable (36,000 new deaths). In the same report, a gradual increase of new cases (2,910 new cases) and deaths (147 new deaths) was reported in Saudi Arabia over this week (1).

On the second of March, Saudi Arabia confirmed (2) its first case of COVID-19, and consequently, a lot of government measures were taken to limit the virus dissemination starting from the closure of Mecca and Medina on 27 February 2020 till home quarantine, travel, and gatherings restrictions and school closures. People have been strictly instructed to maintain social distance, wear a mask, and sanitize their hands frequently (3). Lockdown was released in Saudi Arabia on the 21st of June 2020.

Widespread outbreaks of infectious disease such as COVID-19 and previous epidemics (severe acute respiratory syndrome [SARS] in 2003 or corona influenza epidemic in 2009) are likely to initiate or aggravate mental health concerns such as insomnia, panic attacks, anxiety, and depression due to the impact of containment measures, lifestyle changes, the uncertainties, and fears of getting infected, alarming statistics, media pressure, and financial hardship resulting in anxiety, stress, and depression (4).

A recently published meta-analysis of 66 studies with 221,970 participants concluded that the prevalence of depression, anxiety, distress, and insomnia was 31.4, 31.9, 41.1, and 37.9%, respectively (5). Similarly, recent studies from Saudi Arabia reported high levels of psychological distress during the lockdown stage of the pandemic (6). However, the magnitude of psychological distress after the relaxation of lockdown restrictions and in the longer term in Saudi Arabia remains unclear. Surveys from adults in different countries indicated higher adverse mental health outcomes associated with the pandemic (7–10). Higher depression and anxiety rates were reported during the post-lockdown period compared to other studies that were mostly conducted in the early stages of the pandemic (11). Common predictors associated with psychological distress among the adult population include being healthcare worker, presence of non-infectious chronic or psychiatric patients, COVID-19 patients, quarantined persons (5), being a woman, having children at home, having lower socioeconomic status, adults under 40 years old, unemployment, and frequent exposure to news regarding COVID-19 (12). In addition, Singles, working for the private sector and smokers, reported having experienced worse mental health outcomes (13, 14).

The COVID-19 pandemic may lead to adverse changes in lifestyle behaviors, such as physical inactivity, smoking, and sleep disturbance due to the application of lockdown or even during COVID-19 exit-strategy where physical distancing measures, mask-wearing, the request to work from home, and pandemic-related psychological distress were still in place. Evidence also concluded that healthy lifestyle behaviors are associated with optimal mental wellbeing among adults (15–17). Numerous studies have depicted a direct link between maintaining physical activity and lower of psychological distress (18, 19). Maintaining sleep quality is crucial for strengthening the immunity (20, 21), hence any sleep disturbances due to COVID-19-pandemic-induced stress, may lead to increase the susceptibility to infection, or compromise recovery. Health behaviors are not independently counted, they cluster together (22, 23). Likewise, clustering of unhealthy behaviors has also been found to have synergistic effects, which means that a combination of risk behaviors is more detrimental to health than would be expected from the individual effects of health behaviors (24).

There is still a non-answered hypothesized question if an individual who participates in composite lifestyle behaviors simultaneously during the COVID-19 pandemic will have better mental health, even if the optimal level of each activity is not achieved. To the best of our knowledge, the prevalence of mental health problems such as depression, anxiety, and stress among Saudi Arabian adults, and their association with composite lifestyle behaviors change from pre-pandemic to 16 weeks after COVID-19 pandemic lockdown release have not been explored altogether so far in Saudi Arabia. Thus, the present research is an attempt to fill this gap so that effective mental and health promotion strategies can be planned by practitioners and policymakers.

## Subjects and Methods

### Study Population

This cross-sectional study was conducted on a national level *via* an online survey between October 10 and 31, 2020. Convenience sampling using mass emailing *via* collaborating authors networks, and social media engagement (WhatsApp and Twitter), and snowball sampling, were used for recruitment. Saudi adults (≥18 years) who were residing in Saudi Arabia were eligible to participate. Using EPI-INFO 2002 software, a minimum required sample of 363 Saudi adults was determined, based on a prevalence rate of 38.3% for depression during the COVID-19 lockdown (6) with a precision of 3% and *CI* of 95%. The research received institutional ethical approval from the Research Ethics Committee of Taif Health Affairs, Ministry of Health, Saudi Arabia (IRB: HAP-02-T-067, Number 388). Online written consent was taken from all the participants before they answered the questions and confidentiality will be assured. The research was conducted in accordance with the Declaration of Helsinki.

### Measures

Participants self-reported demographic, medical, weight and height information and completed validated questionnaires relating to mental health (Depression Anxiety and Stress Scale-21 [DASS-21] Arabic version) (25), PA (the International Physical Activity Questionnaire: Short Form [IPAQ-SF]) (26) and described their Sleep quantity and Sleep quality. The history of smoking cigarettes or other tobacco products was also assessed. Further assessments included financial distress during pandemic, previous infection, or exposure to people infected with COVID-19 and COVID-related stigma. Participants were also asked to measure their weight and height. All measures were assessed 16 weeks after the lockdown release except for the four lifestyle behaviors measures (smoking, PA, sitting, and sleep), which also captured pre-COVID-19 restriction information.

### Mental Health

The DASS-21 is a valid and self-report questionnaire used to assess mental wellbeing. We used the validated Arabic version. It consists of three 7 Likert-scaled items for depression, anxiety, and stress each with a 0–3 ordinal scales to describe symptom severity. The participants were asked to indicate the frequency with which the specific emotion had been felt over the past week. Abnormal scores and severity ratings of depression, anxiety, and stress were mentioned elsewhere (25). Higher scores demonstrate the poorer mental health.

### Physical Activity and Sitting Time

The IPAQ-SF has been found to be a reliable measure that has been validated in several countries (*r* = 0.67 and *rho* = 0.77–1.00) and it is acceptable for assessing PA across the various age groups (e.g., 18–70 years). The IPAQ-SF was used to measure the levels of PA and sitting time, and responses were measured using the November 2005 scoring protocol. The IPAQ-SF results were reported as low-, moderate-, or high-PA levels and continuous total metabolic equivalents (METs) minutes per week. Sitting times were reported as minutes per day. Changes in PA and sitting time were reported as no change, positive change, or negative change (increases or decreased) (26–28).

### Sleep

Sleep quality and average hours of sleep duration per night were assessed. Five response options questions ranged from “am sleeping currently much better than usual” to “am sleeping currently much worse than usual” were used to assess sleep quality (29).

### Composite Lifestyle Index

Four lifestyle behaviors were selected for inclusion in this study (i.e., physical activity, sleep, sitting, and tobacco use) and converting the raw value to an index value. Sleep, sitting, and tobacco use behaviors were dichotomized into healthy (low risk) and unhealthy (high risk) categories and scored 1 and 0, respectively. Physical activity was categorized into three risk levels. Summing the scores of the four behavior was done, and change was calculated to reflect a composite lifestyle index (CLI) change score (30). Smoking status of each participant was dichotomized into healthy category (lower-risk = 1 = non-smoker) or unhealthy smoking category (higher-risk = 0 = at least one cigarette per day for at least a month or 0 = a current smoker) (31). Daily sitting time was classified into 2 categories lower-risk (1 ≤ 8 sitting hours per day) and higher-risk categories (0 = ≥8 h per day), as these thresholds of sitting have previously been employed to demonstrate the associations between sitting time and risk of all-cause mortality (32). According to the National Sleep Foundation guidelines, the optimal amount of sleeping is 7–9 h per night for 18–64-year-olds and 7–8 h per night for over 64-year-olds. Sleeping < 7 or >8 h per night is linked to increase the risk of cardiovascular disease and all-cause mortality. Sleep duration was also dichotomized into lower-risk (1 = meeting sleep recommendations) and higher-risk (0 = less than or more than sleep recommendations) (33). Consistent with previous research, PA was categorized into 3 categories lower-risk (2 = high levels of physical activity), moderate risk (1 = moderate levels of physical activity), and higher-risk (0 = low levels of physical activity) physical activity levels (30, 34).

### Statistical Analysis

Statistical analyses were performed using IBM (SPSS) Statistics Version 24.0^*^ software programs. The descriptive statistics, such as frequencies and percentages, were used for categorical variables; means and *SD* or Median and range were used for continuous variables after determining the normality using Shapiro test. The rate of healthy and unhealthy dichotomies was calculated for each lifestyle behavior and the rate of the sample engaging in zero to four healthy lifestyle behaviors were also calculated. Body mass index (kg/m^2^) was computed based on the given weight and height and classified according to the WHO guidelines (35). Reliability was assessed using the Cronbach's Alpha test which resulted in α = 0.948 for the used questionnaire (36).

McNemar-Bowker test was conducted to test the habitual percent change before COVID-19 pandemic and after lockdown release, while Wilcoxon signed-rank test used for comparing the continuous variables. Chi-square test was used to stratify the depression, anxiety, and stress according to the composite behavior change. Three logistic regression models for each psychological distress by categorizing them as yes/no (dummy variable) were conducted to determine the significant contributors associated with depression, anxiety, and stress. For all statistical tests, a significance level was determined below 5% and quoted as two-tailed hypothesis tests.

## Result

The characteristics of the study participants were presented in Table 1. In total, 363 people (mean age 36.26 ± 8.54 years, majority were woman, 65.6%) completed the survey. Most (*n* = 296, 81.5%) were married, and living in mecca (*n* = 253, 69.7%). Most of the participants were university educated (*n* = 235, 64.7%), and reported working in governmental sector (*n* = 222, 61.2%). Almost half of the sample reported diminish in their income (*n* = 186, 51.2%) and a third of the sample (*n* = 121, 33.3%) suffered from the financial distress during the COVID-19 pandemic. Chronic illness was reported in 21.2% of the sample. Sixteen participants (4.4%) had a history of psychiatric illness and 65 participants (17.9%) reported familial history of psychiatric diseases. Regarding the COVID-19 infection status, 17 candidates reported past infection, and more than a one-quarter (*n* = 100, 27.5%) were in direct contact with either infected or suspected case. Thirty-nine participants (10.7%) were suffered from the COVID-related stigma. Computing body mass index revealed that 40.2% of the participants were overweight and 33.1% of the participants were obese. By measuring the DASS 21 scales, 62.5, 83.5, and 73.3% of participants reported normal depression, stress, and anxiety, respectively; however, there is non-neglected percentage that showed a variable range of psychological distress.

**Table 1:** Characteristics of the study participants.

**Characteristic**	**N** **(***n*** = 363)**	**Percentage** **or Mean (SD)** **(Range)**
**Age**	363	36.26 (8.54) (20–59)
**Sex**
Male	125	34.4%
Female	238	65.6%
**Marital status**
Single	53	14.6%
Married	296	81.5%
Divorced	14	3.9%
**Place of residence**
Mecca region	253	69.7%
Riyadh region	36	9.9%
Eastern region	74	20.4%
**Level of education**
Higher education	75	20.7%
University	235	64.7%
Secondary	47	12.9%
Primary	6	1.7%
**Occupation**
Governmental employed	222	61.2%
Private Sector employee	28	7.7%
Freelancer	7	1.9%
Student	22	6.1%
Unemployed/housewife/ Retired	84	23.1%
**Body Mass Index (Kg/m** ^**2**^ **) categories**
Normal weight	93	25.6%
Underweight	4	1.1%
Overweight	146	40.2%
Obese	120	33.1%
**Income change after lockdown release**
Income increased	50	13.8%
Income decreased	186	51.2%
No change	127	35.0%
Financial distress after lockdown release	121	33.3%
Chronic disease status	77	21.2%
History of psychiatric illness	16	4.4%
Infection by COVID- 19	17	4.7%
**DASS 21 results**
Depression	136	37.5%
Anxiety	97	26.7%
Stress	60	16.5%

The habitual changes before and during COVID-19 pandemic showed significant difference and their data described in detail and summed up in Tables 2, 3. The percent changes in composite health behavior score, stratified by depression, anxiety, and stress severity, are illustrated in Table 4. For depression, the composite behavior change did not significantly affect the different strata. On the other hand, stress and anxiety scales exerted a significant difference based on the composite behavior change during pandemic. The significant contributing factors of depression, stress, and anxiety were illustrated using adjusted *ORs* in Table 5. Participants who were highly educated [adjusted *OR* = 2.076, 95% *CI* = (1.012–4.260)], had their private business [adjusted *OR* = 4.345, 95% *CI* = (1.695–11.141)], had a history of psychiatric illness [adjusted *OR* = 3.183, 95% *CI* = (1.026–9.871)], suffered sort of financial distress [adjusted *OR* = 1.777, 95% *CI* = (1.074–2.940)] were more likely to develop depression. The striking finding that participants infected with COVID-19 showed a significant preventive indicator against the depression [adjusted *OR* = 0.105, 95% *CI* = (0.013–0.834), *p* < 0.05]. Participants reported high body weight (obesity, >30 kg/m^2^) [adjusted *OR* = 2.063, 95% *CI* = (1.133–4.122)], had a history of psychiatric illness [adjusted *OR* = 3.288, 95% *CI* = (1.084–9.970)], suffered sort of financial distress [adjusted *OR* = 2.745, 95% *CI* = (1.629–4.627)], and those reported negative composite behavior change [adjusted *OR* = 2.015, 95% *CI* = (2.176–3.453)] were more likely to develop anxiety. Men [adjusted *OR* = 2.770, 95% *CI* = (1.301–5.898)], singles [adjusted *OR* = 5.279, 95% *CI* = (2.179–12.788)], participants with a history of psychiatric illness [adjusted *OR* = 7.352, 95% *CI* = (2.155–25.077)], and those suffered from financial distress [adjusted *OR* = 3.929, 95% *CI* = (2.00–7.717)] were more likely to develop stress.

**Table 2:** Changes in physical activity status, routine sitting, and sleep hygiene: before pandemic vs. after lockdown release.

	**Before pandemic** **number (%)** **(n=363)**	**After lockdown** **release** **number (%)**	**Habitual changes**	***p***
**Overall physical activity level**				
•Low	226 (62.3%)	231 (63.6%)		0.056^a^
•Moderate	136 (37.5%)	124 (34.2%)		
•High	1 (0.3%)	8 (2.2%)		
**Total estimated physical activity minutes per week**				
Median	380	320		0.077^b^
(Range)	(0–3,600)	(0–5,040)		
**Moderate physical activity / wk**				
None	127 (35.0%)*	99 (27.3%)*		0.000^*a^
One day per week	28 (7.7%)	38 (10.5%)	**++ No of Participation in**	
Two days per week	42 (11.6%)*	82(22.6%)*	**Moderate Physical activity**	
Three days per week	70 (19.3%)*	47(12.9%)*	No change: 198 (54.5%)*	
Four days per week	33 (9.1%)	20 (5.5%)	Positive change: 91 (25.1%)*	
Five days per week	26 (7.2%)	26 (7.2%)	Negative change: 74 (20.4%)*	
Six days per week	12 (3.3%)	15 (4.1%)		
Daily	25 (6.9%)	36 (9.9%)		
**Moderate physical activity / day**				
None	127 (35%)*	99 (27.3%)*	No change: 175 (48.2%)*	0.000^*a^
10–20 min/ day	36 (9.9%)*	62 (17.1%)*	Positive change: 117 (32.2%)*	
>20–30 min/ day	80 (22.0%)	71 (19.6%)	Negative change: 71 (19.6%)*	
>30–60 min/ day	69 (19.0%)*	86 (23.7%)*		
More than 1 h/ day	51 (14.0%)	45 (12.4%)		
**Heavy physical activity / wk**				
None	205 (56.5%)	205 (56.5%)		0.039^*a^
One day per week	73 (20.1%)*	59 (16.3%)*	**Fixed Participation in Moderate**	
Two days per week	30 (8.3%)	24 (6.6%)	**Physical Activity**	
Three days per week	28 (7.7%)*	36 (9.9%)*	No change: 304 (83.7%)	
Four days per week	11 (3.0%)	13 (3.6%)	Positive change: 46 (12.7%)	
Five days per week	10 (2.8%)	14 (3.9%)	Negative change: 13 (3.6%)	
Six days per week	2 (0.6%)	6 (1.7%)		
Daily	4 (1.1%)	6 (1.7%)		
**Heavy physical activity / day**				
None	**205 (56.5%)**	**205 (56.5%)**		0.001^*a^
10–20 min/ day	56 (15.4%)*	45 (12.4%)*	No change: 320 (88.2%)	
>20–30 min/ day	37 (10.2%)*	49 (13.5%)*	Positive change: 28 (4.1%)	
>30–60 min/ day	50 (13.8%)	51 (14.0%)	Negative change: 15 (4.1%)	
More than 1 hour/ day	15 (4.1%)	13 (3.6%)		
**Routine sitting in the day**				
1–2 h/ day	74 (20.4%)*	47 (12.9%)*	No change: 165 (45.5%)	0.000*a
3–4 h/ day	98 (27.0%)*	50 (13.8%)*	Decrease in sitting hrs: 30 (8.3%)	
5–6 h/ day	78 (21.5%)	66 (18.2%)	Increase in sitting hrs: 168 (46.2%)	
More than 6 h/ day	113 (31.1%)*	200 (55.1%)*		
Sleep hygiene:				
**A-sleep quantity**				
- less than 7 h	133 (35.5%)	105 (28.9%)	No change: 195 (53.7%)	0.006^*a^
−7–9 h	201 (55.5%)*	167 (46.0%)*	Decrease in sleeping hrs: 45 (12.4%)	
- More than 9 h	29 (9.0%)*	91 (25.1%)*	Increase in sleeping hrs: 123 (33.9%)	
**B-Sleep quality after lockdown release**				
Much better than usual	56 (15.4%)			0.000^*c^
Better than usual	55 (15.2%)			
As usual	176 (48.5%)			
worse than usual	53 (14.6%)			
Much worse than usual	23 (6.3%)			

a *Significant change before COVID-19 pandemic and After lockdown release was assessed using McNemar-Bowker test.

b *Significant change before COVID-19 pandemic and After lockdown release was assessed using Wilicoxon signed rank test.

c *Significant change before COVID-19 pandemic and After lockdown release was assessed using Chi-square test.

*Bolded information denotes the main habitual changes*.

**Table 3:** Changes in smoking habit and total composite behavior score: before pandemic vs. after lockdown release.

	**Before pandemic** **number (%)** **(***n*** = 363)**	**After lockdown** **release** **number (%) (***n*** = 363)**	**Habitual changes**	***p***
**Smoking Habit:**				
**Non-smoking or Ex-smoking**	267 (73.6%)*	275 (75.8%)*	Decrease in the no of smokers with reported 8 cases as Ex-smoker (8.3% of smokers (All were smoking shisha))	0.001*
**Smoking**	96 (26.4%)*	88 (24.2%)*		
*Smoking rate/day				
**- Cigarettes only:**	39 (10.7%)	45 (12.4%)	Negative change in the total number	0.276
Less than 5 times	11 (3.03%)	14 (3.8%)	3 cases (7.7 %) showed a decrease in	
5–10 times	7 (1.9%)	9 (2.4%)	smoking rate **(Negative Change)**	
11–19 times	17 (4.6%)	20 (5.5 %)	While 5 cases (12.8 %) showed an	
20 times and more	4 (1.1%)	2 (0.55%)	increase in the smoking rate	
**-Electronic Cigarettes:**	7 (1.9%)	7 (1.9%)	No change in the total number, but 1	NA
Less than 5 times	2 (0.55%)	2 (0.55%)	case (0.25%) showed an increase in	
5–10 times	2 (0.55%)	1 (0.25%)	the smoking rate **(Negative Change)**	
11–19 times	1 (0.25%)	2 (0.55%)		
20 times and more	2 (0.55%)	2 (0.55%)		
**-Shisha only:**	31 (8.5%)	23 (6.3%)	Change in the total number;	0.007*
Less than 5 times	28 (7.7%)	16 (4.4%)	8 cases became ex-smokers	
5–10 times	0 (0.00%)	3 (0.83%)	**(Positive Change)**	
11–19 times	3 (0.83%)	1 (0.27%)		
20 times and more	0 (0.00%)	3 (0.83%)		
**-Tnbak:**	4 (1.1%)	4 (1.1%)	**No Change**	NA
Less than 5 times	0 (0.00%)	0 (0.00%)		
5–10 times	4 (1.1%)	4 (1.1%)		
11–19 times	0 (0.00%)	0 (0.00%)		
20 times and more	0 (0.00%)	0 (0.00%)		
**Both Cigarettes and Shisha**	15 (4.1%)	9 (2.5%)	Change in the total number;	0.010*
Less than 5 times	9 (2.47%)	3 (0.82%)	6 cases stopped shisha but	
5–10 times	0 (0.00%)	1 (0.27%)	changed to smoke cigarettes only	
11–19 times	4 (1.10%)	1 (0.27%)		
20 times and more	2 (0.55%)	4 (1.10%)		
**Composite Behavior Score**				
0–1 Healthy Behavior	70 (19.3%)*	118 (32.5%)*	No change: 176 (48.5%)	0.000*a
2–3 Healthy Behaviors	236 (65%)^*^	213 (58.7%)*	Negative change: 131 (36.1%)	
4–5 Healthy Behaviors	57 (15.7%)*	32 (8.8%)*	Positive change: 56 (15.4%)	

* *Significant change before COVID-19 pandemic and After lockdown release was assessed using McNemar-Bowker test*.

a *#x0002A;Significant change before COVID-19 pandemic and After lockdown release was assessed using McNemar-Bowker test*.

*NA: Not applicable to conduct the statistical test due to equal proportional distribution*.

*Bolded information denotes the main habitual changes*.

**Table 4:** Influence of the composite health behavior percent change on the depression, anxiety and stress scales.

	**Composite behavior change**			**Statistical test** **(***p***)**
	**Negative change** **(***n*** = 131)**	**No change** **(***n*** = 176)**	**Positive change** **(***n*** = 56)**	
**Depression categories:**				
Normal	79 (60.3%)	113 (64.2%)	35 (62.5%)	
Mild	19 (14.5%)	30 (17.0%)	12 (21.4%)	X^2^ = 10.58
Moderate	23 (17.6%)	22 (12.5%)	9 (16.1%)	0.225
Severe	0 (0.0%)	3 (1.7%)	0 (0.0%)	
Extremely Severe	10 (7.6%)	8 (4.5%)	0 (0.0%)	
**Anxiety categories:**				
Normal	85 (64.9%)^a^	135 (76.7%)^a^	46 (82.1%)^a^	
Mild	25 (19.1%)^a^	17 (9.7%)^a, b^	1 (1.8%)^b^	X^2^ = 20.98
Moderate	9 (6.9%)^a^	11 (6.2%)^a^	8 (14.8%)^a^	0.008*
Severe	3 (2.3%)^a^	4 (2.3%)^a^	1 (1.8%)^a^	
Extremely Severe	9 (6.9%)^a^	9 (5.1%)^a^	0 (0.0%)^a^	
**Stress categories:**				
Normal	108 (82.4%)	149 (84.7%)	46 (82.1%)	
Mild	10 (7.6%)	9 (5.1%)	2 (3.6%)	X^2^ = 14.49
Moderate	1 (0.8%)	9 (5.1%)	3 (5.4%)	0.046*
Severe	5 (3.8%)	4 (2.3%)	5 (8.9%)	
Extremely Severe	7 (5.3%)	5 (2.8%)	0 (0.0%)	

* *Significant composite behavior change was assessed using Chi-square test and Superscripts [^a^] and [^b^] summaries the pairwise comparison*.

**Table 5:** Logistic regression models to identify the significant contributing factors in psychological distress.

	**Variables**	**B**	**Adjusted OR**	**95% CI for AOR**		***p*** **-value**
				**LL**	**UL**	
**Depression**	**Education**					
	University	0.730	2.076	1.012	4.260	0.046*
	Secondary school or lower	0.227	1.254	0.488	3.222	0.638
	**Occupation**					
	Private sector Freelancer Student Unemployed or retired	1.469 1.522 0.103 0.433	4.345 4.583 1.109 1.542	1.695 0.750 0.364 0.806	11.141 28.015 3.381 2.947	0.002* 0.099 0.856 0.191
	**Psychiatric Illness**	1.158	3.183	1.026	9.871	0.045*
	**Financial distress**	0.575	1.777	1.074	2.940	0.025*
	**COVID Infection**	−2.256	0.105	0.013	0.834	0.033*
**Anxiety**	**BMI categories**					
	Underweight Overweight Obese	0.724 0.609 1.771	5.887 1.839 2.063	0.648 0.931 1.133	53.311 3.633 4.122	0.115 0.079 0.04*
	**Psychiatric Illness**	1.190	3.288	1.084	9.97	0.035*
	**History of chronic diseases**	0.372	1.45	0.806	2.609	0.215
	**Financial distress**	1.010	2.745	1.629	4.627	<0.001*
	**Composite Change**					
	Positive Change Negative Change	−0.236 2.701	0.790 2.015	0.349 2.176	1.787 3.453	0.571 0.011*
**Stress**	**Sex**					
	Male	1.019	2.770	1.301	5.898	0.008*
	**Marital status**					
	Single Divorced	1.664 0.194	5.279 1.214	2.179 0.138	12.788 10.655	<0.001* 0.861
	**Psychiatric Illness**	1.995	7.352	2.155	25.077	0.001*
	**Financial distress**	1.368	3.929	2.00	7.717	<0.001*

Depression as dependent variable; X^2^= 58.594; p < 0.001*; Nagelkerke R^2^ = 0.203; Significant predictors in the model: Education (Reference: Higher education), Occupation (Reference: Governmental Sector), Psychiatric Illness/ Financial Distress/ COVID Infection (Reference: No).

Anxiety as dependent variable; X^2^= 38.518; p < 0.001*; Nagelkerke R^2^ = 0.147; Significant predictors in the model: BMI categories (Reference: normal weight), History of chronic diseases/ Psychiatric Illness/ Financial Distress/ Composite Change (Reference: No).

Stress as dependent variable; X^2^= 68.024; p < 0.001*; Nagelkerke R^2^ = 0.289; Significant predictors in the model: Sex (Reference: Female), Marital Status (Reference: Married), Psychiatric Illness/ Financial Distress (Reference: No).

## Discussion

In the current study, we examined the impacts of COVID-19 on lifestyle behavior before the start of the crisis, and 16 weeks after the relaxation of the measures among different regions in Saudi Arabia and studied their reflective influence on the mental health. Depression, stress, and anxiety were reported in 37.5, 26.7, and 16.5% of the participants, respectively, while the majority of the adults were free and mentally stable. This finding was coherent with the recently published Saudi Arabia studies which reported high rates of depression and anxiety related to the COVID-19 pandemic, and lockdown as well (6, 9, 14). The estimated mean scores for depression, anxiety, and stress in our study are all substantially lower than those reported in the Australian survey (29). Moreover, in a national USA study, the results revealed higher rates of psychological distresses among adults during post-lockdown period compared to earlier studies during the pandemic (11). However, numerous studies reported that at the beginning of the pandemic, a large percent reported deterioration in their mental wellbeing psychometrics and afterward during the pandemic concluded that mental health of adults improved as the pandemic persists and the estimated mean scores for depression, anxiety, and stress are mostly within the normal to mild range (13, 37). These differences may be accounted to the data collection time, study tool, discrepancy in government responses to the pandemic, and variability in the population resilience. With the spread of COVID-19, more governmental attention should be paid to its potentially harmful effects on the mental health as recommended by WHO guidelines (38).

The most important finding in our study is that all the studied lifestyle behaviors showed a significant negative change in their pattern before the pandemic and after the lockdown release, both independently and as a composite score, except for smoking. These manifested negative lifestyle changes were significantly associated with anxiety and stress, as symptom severity increased from normal to severe, so did negative changes in composite health behavior change score in contrast to the depression. As many studies significantly linked COVID-19 with developing adverse changes in health behaviors, such as general daily activity, physical activity, sleep hygiene, smoking, and alcohol use (22–24); health-promoting behavior is encouraged to strengthen the immunity against the COVID infection. There is an obvious positive change in the smoking habit, this may be attributed to the nature of COVID-19 infection as a respiratory illness, and smokers are more susceptible to respiratory tract infections. In Saudi Arabia, during pandemic and even after the lockdown release, the cafes that are designated for smoking especially shisha were closed due to the precautionary measures; this might have a positive impact on smoking habit. The pattern of moderate and heavy physical activity showed significant changes before pandemic and after lockdown, although the estimated total average physical activity did not significantly differ (380, 320 min/week, respectively). This is coherent to the recent Australian Survey, showing that Australians aged 15 and over reported 42 min of daily activity, or 294 min/week on average (39). The reported overall decline in physical activity is likely a consequence of the closure of usual exercise venues, given that, here we presented total physical activity, not moderate or moderate-to-vigorous activity. For the sleep hygiene, there is an increase in the number of sleeping hours with a corresponding decrease in the sitting hours per day. Sleep quality showed different varieties among the respondents with about half reported there is no change. This is unsurprising given the potential for psychological distress during an unexpected global pandemic and the variability in the individual response (21). Nonetheless, given the established benefits of health-promoting activities on psychological distresses (18), national implementing strategies are needed.

While studying the causes beyond depression, anxiety, and stress among the suffering participants, logistic regression revealed that financial distress and history of psychiatric illnesses were the common significant increasing factors. Higher education, private work, and self-business were positively associated with those having depression. Surprisingly, COVID infection found as a preventive factor against depression; this hypothesis was previously discussed by Wood et al. study that proved a positive correlation between mental wellbeing and COVID building psychological resilience. Single men were found to be more prone to develop stress; these reported higher levels may be due to the fear from the pandemic consequences, especially regarding job losses and economic stress. Many research reports have highlighted the need for rapid and comprehensive responses to highlighting the mental health not only during the current pandemic, but also, this support will need to be sustained for many years to overcome the COVID-19 mental health consequences (11, 13, 40).

Negative composite lifestyle changes, obesity, as well as a history of chronic diseases positively increase the possibility of developing anxiety. High-risk individuals such as those with chronic diseases and obese may spend a great deal of time thinking and worrying about being infected, this may be a possible explanation of our results. These findings were consistent with those of a previous study in China (41). Our findings were also consistent with Antunes et al. study that concluded that diminish in physical activity with the sedentary behaviors during lockdown were associated with higher levels of anxiety during the COVID-19 pandemic (42).

There are a number of strengths in this study, such as the inclusion of an appropriate sample size, covering multiple health behaviors, and the timing of data collection relative to lockdown restrictions in Saudi Arabia. On the other hand, there are also some limitations to consider. Firstly, the collected data are self-reported which may involve recall bias. Secondly, data are based on a cross-sectional and, therefore, causality cannot be inferred. Finally, our sample was recruited conveniently and consequently generalizability to other populations needs to be confirmed. Longitudinal studies with larger sample sizes are recommended to tackle the changes over time and to assess the impact of lifestyle changes on both physical and mental wellbeing.

## Conclusion

In conclusion, our study exerted a high prevalence of COVID-19-associated adverse psychiatric symptoms, which were reported among the Saudi Arabian adults after the lockdown release. The composite lifestyle score got worse except for smoking among our studied Saudi adults. Financial distress and history of psychiatric illnesses were common significant increasing factors. On the other hand, COVID infection is found as a preventive factor against depression. Negative composite lifestyle changes, obesity, and history of chronic diseases positively increase the possibility of developing anxiety. Effective health promotion strategies directed toward adopting and maintaining positive change in the composite health behaviors should be implemented and evaluated.

## Data Availability Statement

The original contributions presented in the study are included in the article/Supplementary Material, further inquiries can be directed to the corresponding author.

## Ethics Statement

The studies involving human participants were reviewed and approved by Research Ethics Committee of Taif Health Affairs, Ministry of Health, Saudi Arabia. The patients/participants provided their written informed consent to participate in this study.

## Author Contributions

NAb, AA, FA, and NAl designed the study proposal and questionnaire and collect the research data. NAb, AA, FA, NAl, and NO shared in article writing. NO analyzed the data and co-wrote the paper. All authors contributed to the article and approved the submitted version.

## Conflict of Interest

The authors declare that the research was conducted in the absence of any commercial or financial relationships that could be construed as a potential conflict of interest.

## Publisher's Note

All claims expressed in this article are solely those of the authors and do not necessarily represent those of their affiliated organizations, or those of the publisher, the editors and the reviewers. Any product that may be evaluated in this article, or claim that may be made by its manufacturer, is not guaranteed or endorsed by the publisher.
